# Social innovation in education: perspectives from Brazil and Mexico

**DOI:** 10.3389/fsoc.2026.1756628

**Published:** 2026-06-01

**Authors:** Karina Maldonado-Mariscal

**Affiliations:** 1 Social Research Center (sfs), TU Dortmund University, Dortmund, Germany; 2 Faculty of Social Sciences, Arts and Humanities, Kaunas University of Technology (KTU), Kaunas, Lithuania

**Keywords:** Brazil, community participation, education, grassroots innovation, Latin America, Mexico, social innovation, social transformation

## Abstract

Social innovation in education remains under-researched despite its growing importance in addressing educational challenges in Latin America. Whilst innovation in education can encompass systemic reforms or incremental classroom changes, social innovation specifically involves novel partnerships, institutions, methods, and collaborative arrangements amongst communities, schools, local governments, and non-governmental organizations, emphasizing societal participation through co-creation processes. This paper addresses the research question: How do social actors construct and sustain social innovations in education through networks, partnerships, and community participation? Drawing on fieldwork conducted in Brazil and Mexico between 2013 and 2014 and a follow-up interview in 2023, this study employs case study methodology examining three long-standing educational innovations: City School-Apprentice and Campos Salles School in São Paulo, Brazil, and the Institute of Educational Innovation in Chiapas, Mexico. The analysis suggests that, in the cases examined, social innovations emerged through iterative processes involving collective action, sustained relationship-building, and collaborative governance at the micro level. The findings show how locally rooted networks developed connections to wider institutional structures, under conditions that varied considerably between the two national contexts. The paper's primary contribution lies in offering an empirically grounded sociological account of social innovation in education in two contrasting Latin American contexts. This study shows the influence of diverse stakeholders on the implementation of educational innovations in environments with limited resources. It also emphasizes the transformative potential of grassroots innovation, civil society involvement, and community-centred approaches to educational change in Latin America.

## 1 Introduction

Despite economic growth in Mexico and Brazil in the 1980s and 1990s, as well as in more recent years, social inequality has remained one of the region's main problems (OECD, 2025a; O'Donnell, 1996), particularly affecting access to quality formal education. This challenge persists, with inequalities in education strengthening in both urban and rural regions. There is an unequal distribution of material resources in public and private schools, and teachers are poorly rewarded (Stromquist, 2004), and student teachers often have very decontextualized teaching experiences (Flores et al., 2023). Furthermore, the education of indigenous and non-indigenous populations remains intrinsic at the systemic level (González Pandiella and and Maravalle, 2024).

The central arguments related to this problem focus on unequal income distribution as well as unequal access to good quality public education. Understanding these challenges requires examining how the concept of innovation in education itself has evolved. The paradigm of innovation has evolved over recent decades worldwide, an historical analysis from 1939 to 2019 demonstrates that whilst innovation has long been primarily associated with technological transformations, it has gradually expanded to include social innovation (Maldonado-Mariscal and Alijew, 2023). This evolution becomes particularly relevant when examining educational systems in Latin America, where social innovation represented a new innovation paradigm in the 2010s, shifting from a market-driven perspective to a social-oriented one (Maldonado-Mariscal, 2023).

In Mexico, for example, like in Brazil, the increase in educational coverage in the last decade has been remarkable (OECD, 2011, 2025b; PISA, 2012a,b); however, the lack of modernization of teacher training reflects the low quality of education (PISA, 2012b) and the differentiated access to good quality educational institutions, due to a significant diversity of private and very high-cost schools. In Mexico, it is also argued that the decentralization of education from the federal government to the states has hardly contributed to the improvement of the quality of education (Robles Peiro, 2006), due to the continued high centralization of salaries and bureaucracies (Di Gropello, 1999, p. 163), maintaining high spending on education but very little of this spending on actual improvement of curricula, teacher training or access to good quality education in rural areas. Current studies show how in Brazil there is high inequality in education spending (Bertoni et al., 2020) funded by local governments, which means that there is a funding gap between high and low income regions of the country, which may contribute to greater inequality in education in this country.

Decades earlier, the emergence of social movements in education in the 1960s in the region, but especially in Brazil, introduces civil society as a new actor in education (da Gohn, 2013; Maldonado-Mariscal, 2020), pointing to innovation in education and a new distribution of power.

The role of civil society, communities, and schools in constructing social innovations becomes particularly significant when examining changes at different levels of the education system. At the micro level, changes occur at the local level with greater involvement of educational and community actors, representing interventions from below (grassroots) that differentiate innovations from top-down reforms (Maldonado-Mariscal and Schröder, 2023). In Brazil, relevant case studies show examples of community participation in education through the creation of new networks and forms of collaboration (Maldonado-Mariscal, 2017).

For this research, the countries of Mexico and Brazil were selected because public spending on educational institutions in both countries is amongst the highest in the Latin American region after Chile. Additionally, both countries show the highest number of innovations in education, according to regional studies on innovation (de Marulanda and Tancredi, 2010; Rodríguez Herrera and Alvarado, 2008). According to these studies, innovation in education in Brazil is especially notable in this region, as Brazil is shown as one of the countries with the highest number of innovations in education amongst 18 Latin American countries in the period 1998–1999, whilst Mexico appears as the country with the second highest number of innovations (Blanco and Messina, 2000; Maldonado-Mariscal, 2017). In this sense, public expenditure on educational institutions in Mexico and Brazil is amongst the highest in the Latin American region after Chile. Whilst in Mexico it was 2.8% as a percentage of GDP in 2017 (OECD, 2020), spending in Brazil in 2015 was 5.5% as a percentage of GDP (OECD, 2018).

This study examined the following research question: *How do social actors drive social innovations in education, and what roles do networks, partnerships, and community engagement play in these innovation processes?* The following Section 2 introduces the theoretical framework, defining social innovation and its links with grassroots innovation within the broader context of innovation studies.

## 2 Defining social innovation education

Research in social innovation shows the need for new perspectives on innovation (Krlev et al., 2020; Howaldt and Schwarz, 2010; Franz et al., 2012), more toward the participation of society, but also with a broader and more systemic perspective (McGowan et al., 2017; Edquist, 2011) including actors from civil society, economy, science, and politics as a system.

There is a growing scientific debate on social innovation in education (Alden Rivers et al., 2015; Loogma et al., 2013; Maldonado-Mariscal and Schröder, 2023). On the one hand, part of the literature asserts that innovation in education is a new technology applied to education (Ferguson et al., 2019; Vincent-Lancrin et al., 2019). On the other hand, other approaches recognize the value of society innovating in education. The latter approach recognizes how innovation is a result of cyclical dynamics and societal change (Maldonado-Mariscal, 2020). This approach explains how new collaborations, new institutions and new actors can emerge in forms of collaboration between communities, schools, local government, and non-governmental organizations (Maldonado-Mariscal, 2024), and how these new networks build an innovation ecosystem where old actors take on new roles (Schröder and Krüger, 2019).

Social innovation education is a field that is still searching for common ground concepts and theoretical understanding (Maldonado-Mariscal, 2024). Whilst some studies understand social innovation education as the use of technology in the classroom (Durando, 2017), other studies understand it as systemic changes (Fullan, 1972), or new governance and organizational change (Cerna, 2014) with strong societal involvement (Maldonado-Mariscal, 2020). Recent research emphasizes that social innovation in education requires more than new technological innovation in the classrooms, new curricula and new educational reforms, but rather a comprehensive understanding of different dimensions and types of social innovation in education (Maldonado-Mariscal and Schröder, 2023).

The field of innovation in education has tended to be understood as pedagogical and technological innovations and to a lesser extent interpreted as social innovations. In education, the most recognized innovations are those promoted by the knowledge systems of the most advanced countries with greater technology and research capacity, but also through innovations that are produced by the use of technologies and new pedagogies (OECD, 2016). In the field of social innovation in education, there are different levels at which innovative action can take place (Maldonado-Mariscal and Schröder, 2023). At the macro level, there is a legislative framework, such as reforms and change in institutional regulations, whilst at the meso level, there is change in educational and learning models or changes in educational structures. At the micro level, changes occur at the local level with greater involvement of educational and community actors, representing interventions from below that differentiate innovations from top-down reforms.

Social innovations in education are complex as they involve specific stakeholders of the educational field, e.g., educational institutions (schools, universities) and various actors (teachers, parents, university professors, communities, NGOs). Both kind of institutions and stakeholders can innovate, often in programmes, projects, methodologies, organization, structures or networks. Furthermore, the main actors involved in social innovation in formal education are usually NGOs, universities, communities, schools, policymakers, and foundations, highlighting the crucial role of civil society in educational transformation. The role of civil society in educational innovation is also crucial for modernizing the formal education system in the sense of changing social practices to solve educational demands and challenges in a broader way, including methods of co-creation of better solutions with different stakeholders, and designing new ways of organization (Maldonado-Mariscal and Schröder, 2023; Bourdieu, 1986). According to (Arefi 2003) social capital can be recognized through networks of “civic engagement and norms of reciprocity” (Arefi, 2003, p. 392) because this allows access to resources from different contacts of the network and booster collective action.

In education it is argued that innovations are shaped by the characteristics of their environment, more specifically, the characteristics of space and time that define social innovations (Mohr, 1978; Mulgan, 2012). (Martin 2010) describes that innovations in education need a long time to show themselves because they produce long-term effects in their adaptation process, and cannot foster better outcomes in the short term. Therefore, there is needed an analysis of social innovation in education with an historical perspective and a relation to their context.

### 2.1 Connecting social innovation in education and grassroots innovation

A theoretical contribution of this study concerns the relationship between social innovation in education and grassroots innovation. Social innovation in education is understood here as

“new social practices in education, new forms of collaboration, the creation of new institutions, and the creation of alliances and networks of different actors who previously did not collaborate with each other,” (Maldonado-Mariscal and Schröder, 2023, p. 222)

emerging within specific geographical and historical contexts (Maldonado-Mariscal, 2020).

The three case studies presented here share important characteristics with grassroots innovation, defined as

“a networks of activists and organizations generating bottom-up solutions that respond to local situations and the interests and values of the communities involved.” (Seyfang and Smith, 2007; Maldonado-Mariscal, 2023)

All three cases originated from civil society initiatives responding to gaps in state provision, were driven by community actors, and developed locally embedded solutions to educational inequalities. This suggests a strong conceptual overlap between social innovation in education and grassroots innovation that has received insufficient scholarly attention. Strengthening this theoretical link offers a promising avenue for future research, particularly in Latin American contexts where grassroots and civil society-led educational initiatives have historically played a significant role in addressing structural inequalities that the state has failed to resolve.

## 3 Method

This study employed a qualitative research design, using comparative case studies of social innovation in education across two countries: Brazil and Mexico. The data collection involved three primary methods: case study analysis, semi-structured interviews, and participant observation. The three cases were selected through a systematic process that combined documentary research, including an extensive online literature review, with cases identified through the Latin American and Caribbean Education Innovation Network “INNOVEMOS,” a UNESCO platform that systematically documents regional educational innovations across Latin America and the Caribbean.

Case selection was further validated through two exploratory field research: the first in Rio de Janeiro, Brazil (in 2013), and the second in Mexico City, Mexico (in May 2014). These fieldwork stages served three purposes: (1) validating the relevance of the pre-selected cases, (2) identifying key actors at the national level who could provide broader perspectives on social innovation in education, and (3) expanding the network of interview contacts through snowball sampling. Within each case study, in-depth interviews and participant observation were conducted. Additionally, a focus group was carried out in one of the two countries (Brazil). A summary of the number of interviews, focus groups, and participant observation sessions is presented in Table 1 below. The full list of interview participants, organized by country and role, is presented in Table 2.

**Table 1:** Overview of the used methods for this research.

Data collection	Case 1 Brazil	Case 2 Brazil	Case 3 Mexico
Interviews	22		15
Focus groups	yes	no	no
Participatory observation	yes	yes	yes

Self-elaborated.

**Table 2:** List of interview participants by country, role, and level.

ID	Country	Role	Level
–Brazil–			
BR-01	Brazil	National Expert (Researcher)	National
BR-02	Brazil	Local / Case Study Specialist	Local — Case 1
BR-03	Brazil	Local / Case Study Specialist	Local — Case 1
BR-04	Brazil	Local / Case Study Specialist	Local — Case 1
BR-05	Brazil	Local / Case Study Specialist	Local — Case 1
BR-06	Brazil	Local / Case Study Specialist	Local — Case 1
BR-07	Brazil	State Expert (Government) — Focus Group (5 participants)	State
BR-08	Brazil	Local / Case Study Specialist	Local — Case 2
BR-09	Brazil	Local / Case Study Specialist	Local — Case 2
BR-10	Brazil	Local / Case Study Specialist	Local — Case 2
BR-11	Brazil	Teacher	Local
BR-12	Brazil	Teacher	Local
BR-13	Brazil	Teacher	Local
BR-14	Brazil	Teacher	Local
BR-15	Brazil	Teacher	Local
BR-16	Brazil	Teacher	Local
BR-17	Brazil	Teacher	Local
BR-18	Brazil	State Expert (Government)	State
BR-19	Brazil	State Expert (Government)	State
BR-20	Brazil	State Expert (Government)	State
BR-21	Brazil	State Expert (Government)	State
BR-22	Brazil	Local Expert (Government)	Local
–Mexico–			
MX-01	Mexico	National Expert (Government & Researcher)	National
MX-02	Mexico	National Expert (Researcher)	National
MX-03	Mexico	State Expert (Government)	State
MX-04	Mexico	State Expert (Researcher)	State
MX-05	Mexico	State Expert (Researcher)	State
MX-06	Mexico	State Expert (Researcher)	State
MX-07	Mexico	Local / Case Study Specialist	Local
MX-08	Mexico	Local / Case Study Specialist	Local
MX-09	Mexico	Local / Case Study Specialist	Local
MX-10	Mexico	Local / Case Study Specialist	Local
MX-11	Mexico	Local / Case Study Specialist	Local
MX-12	Mexico	Local / Case Study Specialist	Local
MX-13	Mexico	Teacher	Local
MX-14	Mexico	Teacher	Local
MX-15	Mexico	Teacher	Local
**Summary**			
	**Mexico**	**15 interviews**	
	**Brazil**	**22 interviews** **+** **1 focus group (5 participants)**	

Following the exploratory field visits, empirical data were collected during two extended periods of fieldwork: a 2-month stay in São Paulo, Brazil (December 2014 to end of January 2015), and a one-month stay in San Cristóbal de las Casas, Chiapas, Mexico (22nd April to 22nd May 2014). To provide an illustrative update on the evolution of networks and partnerships in educational innovation, a follow-up online interview was conducted via Zoom in May 2023 with a Brazilian researcher who had been interviewed during the original data collection and who holds an expert overview of the educational innovation landscape in the country. It should be noted that this follow-up was limited to the Brazilian case and constitutes a partial update rather than a systematic data collection effort. For the Mexican case, email correspondence was maintained to gain further insight into the current state of the case studies. The exchange offered indicative insights into how the networks established by civil society organizations in the 1990s and 2000s may have evolved.

The following paragraphs describe the processes involved in each stage of the fieldwork, covering the three case studies, a total of 37 interviews, five participant observations, and one focus group comprising five participants.

### 3.1 Case studies

The case studies were initially identified through the INNOVEMOS platform, as described above. Selection was based on the following criteria: (a) the educational innovation must have been in operation for at least 20 years; (b) the innovation must have achieved acceptance amongst its participants; and (c) the innovation must have been fully implemented, so that a meaningful analysis of the case could be conducted.

Case studies were employed as the primary method to document and understand in depth the nature of each innovation, its context, the actors involved, and the networks built through the innovation process. Three case studies were selected across the two country contexts: two in Brazil, located in São Paulo, and one in Mexico, located in Chiapas. The cases were chosen to enable comparison across distinct socio-spatial contexts: the City-School Apprentice (Case 1), situated in the city centre of São Paulo; the Campos Salles School (Case 2), located in the urban periphery of São Paulo; and the House of Science and Institute of Educational Innovation (Case 3), based in the urban-poor region of San Cristóbal de las Casas, Chiapas. Together, these cases provided a basis for national comparison between two distinct educational and social contexts.

### 3.2 Interviews

In-depth interviews were conducted to collect detailed individual perspectives and experiences related to the development, uptake, and implementation of social innovations in education. A total of 37 interviews were conducted between 2013 and 2014, of which 22 were carried out in Brazil and 15 in Mexico. An additional online interview was conducted in May 2023 to provide an illustrative update on the evolution of the cases studied.

The interviews were designed as semi-structured interviews and tailored to four categories of stakeholders: national experts, regional experts, local and case study related experts, and teachers. Topics covered included the formal education system and aspects of inequality, biographical narratives related to change and innovation, innovation practices and autonomy, perceptions of innovation in education, obstacles and drivers of innovation, the role of the teacher, and attitudes toward alternative educational programmes. Teachers were additionally asked about their own experience as educators and their understanding of what innovation means in their specific context. All interviewees were informed of the purpose of the research and asked for their consent prior to participation.

Interview partners were selected purposively and complemented through snowball sampling, whereby interviewees were asked to suggest further contact persons involved in the case studies. Each interview was conducted in person and voice recorded. Transcriptions were made in the original language, Spanish or Portuguese, and each transcript was reviewed for accuracy before analysis began.

The analysis followed a hybrid approach to qualitative content analysis (Mayring, 2000). An initial set of deductive categories was derived from the theoretical framework guiding the study, reflecting key concepts such as autonomy, power, and educational innovation. A subsequent inductive coding pass allowed additional categories to emerge directly from the interview material, ensuring that participant perspectives not anticipated by the framework were captured. Data collection and coding continued until thematic saturation was reached, meaning that no new categories or patterns were identified across successive interviews.

All transcripts were analyzed using Atlas.ti through a two-cycle coding process with categories manually coded and highlighted. In the first cycle, deductive codes were applied based on the predefined category system. In the second cycle, open coding was used to identify residual or emergent themes. Code co-occurrence and frequency were subsequently examined to support case comparison, allowing patterns and divergences across stakeholder groups and national contexts to be systematically identified.

The final analytical categories were: biographical details of the interviewee; knowledge of the national education system and its contradictions; key actors within the system; the dimensions of power, autonomy and inequality; perceptions of educational innovation; changes observed in the last 5 to 10 years; elements of innovation within the local education system; and teachers' roles in innovation. Triangulation was achieved by including multiple stakeholder perspectives, such as national, regional, local, and classroom-level, to ensure that findings were cross-checked across different positions within the education system. The analytical categories comprised: biographical details of the interviewee; knowledge of the national education system and its contradictions; key actors in the education system; dimensions of power, autonomy, and inequality; perceptions of innovation in education; changes observed over the last 5 to 10 years; elements of innovation in the local education system; and the role of teachers in innovation.

### 3.3 Participant observations

Participant observations were carried out with the aim of collecting data in the natural context of the actors and documenting their behaviors as they occurred. A total of five observation sessions were conducted across different settings, including: preparatory meetings amongst teachers within one of the case study innovations; the first day of class in one of the innovation case studies (2015); meetings between parents and the leader of one of the educational innovations; meetings between one of the case study innovations and its partner organizations; and meetings involving teachers, coordinators, and representatives of the Municipal Secretariat of Education of São Paulo. Observations were documented in the form of research notes and research diaries, serving to complement and contextualize the data gathered through the case studies and in-depth interviews.

### 3.4 Focus group

One focus group was conducted in Brazil, comprising five participants from the Regional State Directorate of Education in São Paulo. The focus group aimed to explore the cultural norms of a regional institution with regard to innovation in education, as well as to gain an overview of the regulatory framework governing educational innovation in the region. The session was voice recorded and subsequently transcribed and analyzed alongside the other data sources.

### 3.5 Rationale

The rationale for conducting this comparative study between Brazil and Mexico stems from several key factors. First, both countries represent significant cases for understanding social innovation in education in Latin America, as they demonstrate the highest number of educational innovations in the region and maintain substantial public investment in education (de Marulanda and Tancredi, 2010; Rodríguez Herrera and Alvarado, 2008). Second, both countries share similar challenges regarding unequal access to quality education and the need for modernization of their education systems, making them suitable for comparative analysis. Third, the selection of long-standing innovations (operating for at least 20 years) allows for the examination of sustained impact, network development, and the evolution of collaborative relationships over time, which is essential for understanding how social actors construct and implement social innovations in education. The decision to focus on civil society organizations, particularly in Brazil, responds to the historical emergence of social movements in education in the 1960s that introduced civil society as a new actor in the educational landscape (da Gohn, 2013; da Gohn and Maldonado-Mariscal, 2023). This focus enables a deeper understanding of how community-led initiatives, grassroots innovation, and bottom-up approaches contribute to educational transformation at the local level with greater involvement of educational and community actors (Maldonado-Mariscal and Schröder, 2023). Furthermore, the study addresses a gap in the literature on social innovation in education, which remains an under-researched field despite its growing importance in addressing educational challenges in Latin America.

Sections 4 and 5 present the main results, organized by case study. Section 4.1 introduces cases of social innovation in Brazil and Section 4.2 introduces the Mexican case study. Section 5 then explains these findings. Section 6 introduces a discussion and the limitations of this study, while Section 7 offers conclusions and an outlook in a broader context.

## 4 Introducing case studies in Brazil and Mexico

### 4.1 Social innovation in education in Brazil

#### 4.1.1 Case 1: City-School Apprentice

This case study examines a Non-Governmental Organisation (NGO) based in the city centre of São Paulo. Cidade Escola Aprendiz (City-School Apprentice in English) is an NGO that started its activities in 1997 (Cidade Escola Aprendiz, 2024). Its founders aimed to strengthen school-community relations and intervene to reduce violence through art, culture, and education in a high-crime neighborhood of São Paulo.

City-School Apprentice was initiated to create links between the community of Vila Madalena and local schools. One of the main objectives was to gain leadership by using public space for educational purposes. For example, they started with educational walks and art interventions in specific areas that were previously problematic due to crime. The organization developed the Bairro-escola (Neighborhood-school) concept, a social practice that proposes a process of shared learning bringing together schools, communities, social organizations, businesses, and public authorities.

The team of this organization highly valued interdisciplinarity, as it consisted of communicators, psychologists, sociologists, and journalists. The main activities were based on communication, education and art programmes to promote citizenship and social cohesion. City-School Apprentice projects aim to develop a learning community, have a strong associative and partnership-building element, as the diffusion of innovation occurs in a network (Medeiros and Galiano, 2005). One of the main innovations of this initiative concerns the strong network developed by the initiative, not only with schools but also with communities, researchers and local government. The main actors involved in this innovative City-School Apprentice initiative were civil society, the public sector and, especially, the community. Subsequently, the private sector, as small donors, supported the initiative and researchers were also involved, developing specific case studies on the impact of City-School Apprentice in Vila Madalena in São Paulo.

#### 4.1.2 Case 2: Campos Salles School

This case study examines a new school model implemented in the community of Heliópolis, a peripheral urban community of São Paulo. The Campos Salles School Project started with the inspiration of a Basic Education School in Portugal, the Escola da Ponte (Bridge-School). This school implemented new principles and methodologies to link school and community in 1976 (Martinho and and Freire da Silva, 2008). The main principles of the Ponte-School in Portugal were to educate with a focus on human rights, equality, and participation of the educational community itself and the pupils.

The new model of the Campos Salles School was initiated in 1995 by studying the experience of the Ponte-School and developing a new school model of its own tailored to the needs of the Heliópolis community. One of the main objectives of this school was to build ownership and leadership in collaboration with the leaders of the Heliópolis community. For example, they created the School Commission of Parents, Students and Teachers, and together with the school approved in 2005 the proposal for the implementation of a new school model in Heliópolis, a Campos Salles community school.

The team consists of a very diverse profile of teachers; however, in this innovation the role of the school principal was crucial for the development and implementation of the new school model at Campos Salles School. One of the main examples of educational social innovation in this case study lies in the physical transformation of the school into a Unified Educational Centre (CEU). The school's infrastructure became the Heliópolis Educational and Cultural Community Centre, a space that today represents a conquest of public space for the community and the school.

The stakeholders in this case study are very diverse. Civil society in the form of the Heliópolis Residents' Association and NGOs supporting the school model were very relevant to the implementation, as well as the school principal. In this case the private sector was also involved, as companies donated computer equipment for the school. In addition, public actors such as the local and regional school administration were also involved as stakeholders who supported the implementation of this new model through the development of individualized assessment software for the specific assessment practices of the pupils of this school. At the time of the original fieldwork in 2014, no formal investigation of the case was being conducted outside of the research undertaken by the author of this work.

Campos Salles School explicitly stated in its Political Pedagogic Project (PPP) of 2014 the high relevance of building social capital in the whole community of Heliópolis:

“Identify the many educational possibilities with a coordination of diverse actors, establishing collective actions and prioritise the permanent formation of its residents, through its associations, entities, projects, etc. for the development of human capital, and the strengthening of social capital of the community.” (Political Pedagogic Project, 2014, p. 50)

The Campos Salles School demonstrates how social innovation in education emerges through the collaboration of multiple actors, including community associations, teachers, school leadership, local government, and civil society organizations. The case illustrates the importance of adapting international educational models to local contexts and the role of community participation in transforming both the physical and pedagogical spaces of education.

### 4.2 Social innovation in education in Mexico

The Mexican context of social innovation in education shows strong roots in social inequality and inequity in education in the country. Experiences of innovation in higher education together with municipal government show especially the development of local networks initiated by universities, supported by local policies and local community actors and the need to implement projects in the field of education. However, some of these initiatives did not achieve institutional sustainability as decentralization of power did not occur and local social capital was not sufficiently mature (Bourdieu, 1986). Chiapas is one of the states which has occupied not only the highest levels of poverty and social exclusion for decades but also the lowest rates of educational attainment amongst the indigenous population. The educational system in Mexico is highly centralized and has high fragmentation in teacher training. In addition, inequalities in education for indigenous and non-indigenous teachers are intrinsic at the systemic level with a strong need for intercultural education (Schmelkes, 2013). Some of the main innovations in southern Mexico focus on the development of alternative education for indigenous communities as autonomous communities in Chiapas. But more and more social innovations in education show the transformative potential build upon civil society (Keck and Vázquez Sánchez, 2022; Villa-Enciso et al., 2023).

At the national level, a research expert in the field of social innovation in education in Mexico recognized the key role of engagement of actors and the difficulty of transferring innovation, since every context embodies different characteristics and problems. She argued:

“I don't believe in innovations. In education it is very difficult to innovate, because you need the people who make the innovations and you can't clone them.” (Interview MX-01).

#### 4.2.1 Case 3: The House of Science and Institute of Educational Innovation (INED)

The Institute of Educational Innovation (INED), in Spanish Instituto de Innovación Educativa, is a Non-Governmental Organisation in San Cristóbal de las Casas in Chiapas. The institution was founded in 1994 under the name The House of Science (Casa de la Ciencia). Initially, its main objective was to create a space to share science with society and local communities, functioning as a kind of science shop. Later, its focus shifted to addressing local needs to improve the quality of compulsory education. This institution focused for many years on improving the education of students. However, its objectives shifted to teacher training after it identified a strong need for in-service training for teachers and teacher educators. This meant that INED evolved from community and school-centred project development to teacher training and educational leadership (Estrada, 2007, 2009). Today, its aim is to contribute to the training of teachers in the region with a broader perspective, incorporating ethical and emotional dimensions and fostering greater autonomy.

The INED project initially focused on school-based interventions but shifted toward teacher training after identifying a lack of state support and insufficient training provision in Chiapas. Its approach to social innovation in education includes the use of participatory methods for diagnosis, decision-making, and training, as well as working with indigenous teachers in multi-grade rural classrooms in southern Mexico. A distinctive feature of this approach is its emphasis on the personal and socio-psychological development of the teacher, recognizing that effective teaching requires self-awareness, an understanding of one's own limitations and strengths, and continuous personal growth (Keck, 2018).

One INED representative reflected on their main aim as the Institute of Educational Innovation:

“We intend to be a centre of educational innovation that links international and national institutions and resources and to be a reflexive and critical space.” (Interview BR-02)

The stakeholders in this case study are also varied. INED was first supported by its own communities and by many volunteers. Once this institution became more recognized locally, it received support from international foundations, such as the Kellogg Foundation and the Ford Foundation, and from the local educational government of Chiapas. The institution has developed a strong network of partners; however, financial support is no longer provided by the local education government of Chiapas, reflecting the challenges of sustainability faced by civil society-led educational innovations in contexts of limited state support.

## 5 Findings

### 5.1 Key actors involved

In the following Table 3 we can observe the participation of different actors, such as researchers, public sector, private sector, civil society, and local communities. This is relevant to understand the composition of the different innovations in education and the formation of networks. In this table we can see that in the case of both the Brazil City-School Apprentice and the INED Institute, all actors such as researchers, the public sector, the private sector and local communities are represented, in contrast to the Campos Salles School, where researchers have not participated.

**Table 3:** Stakeholders of social innovation in education: case studies in Mexico and Brazil.

Stakeholders of social innovation in education	(1) City-School Apprentice (Sao Paulo, Brazil)	(2) Campos Salles School (Sao Paulo, Brazil)	(3) Institute of Educational Innovation (Chiapas, Mexico)
Researchers	yes	no	yes
Public sector	yes	yes	yes
Private sector	yes	yes	yes
Civil society	yes	yes (school+ community)	yes (foundation+ teachers)
Local communities	yes	yes	yes

Own elaboration based on fieldwork data.

As researchers, we refer to the involvement of scientists in the documentation or monitoring of selected cases. The public sector is represented by the government and different ministerial agencies at local, regional, and national levels. The private sector refers to companies and foundations that support innovation, usually in the form of funding or resource provision. Civil society refers to organized civil society institutions, such as NGOs and associations, which participated in the innovation or its activities. Finally, local communities refer to community associations, residents' organizations or grassroots institutions that are actively involved in the innovation.

Table 3 illustrates the participation of different actors across the three case studies: researchers, the public sector, the private sector, civil society, and local communities. This is relevant for understanding the composition of the different social innovations in education and the formation of networks that enable and sustain these innovations.

#### 5.1.1 Similarities and differences in stakeholder participation

The analysis reveals important differences in actor constellations. Both City-School-Apprentice and INED involve all actor categories, suggesting these innovations benefit from diverse sources of knowledge, legitimacy, and resources. In contrast, Campos Salles School engaged all actors except researchers, indicating that this innovation developed primarily through the collaboration of educational practitioners, community members, and institutional actors without formal academic involvement during the study period.

Civil society organizations and local communities appear as active participants in all three cases, underscoring their crucial role as drivers and facilitators of educational innovation. This reflects the emphasis on community participation and co-creation that characterizes social innovation in education.

The involvement of the public sector in all cases demonstrates that even grassroots-initiated innovations require engagement with formal educational institutions and government actors to achieve implementation and sustainability.

Private sector participation, whilst present in all cases, takes different forms. In City-School-Apprentice and Campos Salles School, it primarily consisted of donations and support, whilst in INED, international foundations such as the Kellogg Foundation and Ford Foundation provided significant financial support. This pattern suggests that private sector engagement in social innovation in education in Latin America often takes the form of philanthropic support rather than commercial partnerships.

Researcher involvement varies significantly. In City-School-Apprentice, researchers from the local university actively collaborate in developing new understandings of education and implementing Working Groups. In INED, they contribute to diagnostic work and experimental projects. Their absence in Campos Salles School does not diminish the innovation's significance but highlights that educational innovation can emerge from practitioner-led initiatives without formal academic partnerships, although such partnerships may develop as innovations gain recognition.

These patterns of actor involvement demonstrate that social innovation in education emerges through various configurations of stakeholders. Civil society organizations and local communities play a central role in all cases, while the combination of other actors varies according to local contexts, institutional arrangements, and the evolution of each initiative over time.

### 5.2 Social innovation in education: a multi-perspective approach

The understanding of (social) innovation in education varies significantly amongst stakeholders, reflecting their distinct positions and priorities within the educational system. Table 4 presents the perspectives of key actors involved in the case studies, including NGO representatives, government officials, school leaders, teachers, and researchers from Brazil and Mexico, drawn from the interview data.

**Table 4:** Stakeholder perspectives on social innovation in education.

Stakeholder type	Country	Understanding of innovation in education
Local / Case Study Specialist (researcher)	Brazil	“Really innovative is what is able to transform this structure (the core structure), what allows to transform the nature of the whole institution”
State Expert (Government), School for Teachers' Formation	Brazil	“For us, innovation is to take the practice of the teacher and develop his continuing education, to break with what is already set – that the knowledge of the schools' environment is out of the school”
State Expert (Government)	Brazil	“Innovation… is change of time and space in the school (…); then, is to think education in the neighbourhood, in the community and to observe what happens out of the school”
Local / Case Study Specialist (School)	Brazil	“Everything is innovative in our project. The physical infrastructure is the most visible. The professors are not alone in a classroom. (…) Everything is discussed and elaborated in a team”
Teacher of 5th Grade	Brazil	“I think ours is an innovative project! Because it proposes solutions to old problems that persist in Brazilian education and it has a social orientation, the school is connected to the community, this school wouldn't exist without the community”
Local / Case Study Specialist	Mexico	“The main elements of innovation here are: the training of educational agents, innovative spaces for teacher training, the inclusion of practical elements, the formality of our work, as we are not there to simulate... We are there to carry out training amongst professionals, to encourage the real participation of those who were in training. This was experienced as very innovative by the teachers”
National Expert (Government & Researcher)	Mexico	“An innovation is a different practice, different from the conventional ones, it is a proposal that develops aspects that have to do with the cultural, historical context, cosmovisions of the social groups and are significant to them, and allows them to develop culturally in educational-formative terms. It is associated with the history and culture of the population it serves”

Source: Own elaboration based on interview data.

The perspectives presented in Table 4 reveal a varied but interconnected understanding of social innovation in education across stakeholders and country contexts.

In the Brazilian cases, innovation is strongly associated with the integration of school and community. Interviewees consistently emphasize that education must be rooted in the neighborhood and social context, challenging the traditional boundaries between school and community. A state government representative underlines that knowledge exists outside the school environment, whilst a teacher notes that the school would not exist without its community. This shared perspective suggests that social innovation in education in Brazil is understood as a process of opening institutions to their social surroundings.

In the Mexican case, innovation is understood as a rupture with conventional practices, combined with the inclusion of cultural traditions and indigenous cosmovisions. A researcher and state representative stress that innovation must be connected to the historical and cultural context of the population it serves, which is particularly relevant in Chiapas, where indigenous communities face significant educational inequalities. An NGO coordinator further emphasizes the transformation of teacher training toward more participatory and contextually relevant approaches.

Three cross-cutting themes emerge from these perspectives. First, innovation is understood as fundamental transformation rather than incremental improvement, whether at the level of institutional structures or everyday practices. Second, collective participation and co-creation are central to how innovation is conceived and implemented across both contexts. Third, understandings of innovation vary by actor position: practitioners focus on concrete changes, whilst researchers and government officials emphasize systemic transformation.

Taken together, these perspectives suggest that social innovation in education is characterized by structural change, community participation, and cultural relevance, emerging from collective action rather than individual initiatives.

### 5.3 Key elements of innovation

#### 5.3.1 Case 1: City-School Apprentice

The NGO's networks and partnerships are particularly strong with the local community and schools, and also demonstrate close collaboration with local government. One of the main partnerships with universities is the collaboration with the University of São Paulo. The university collaborates with City-School-Apprentice in building a new understanding of education through the development and implementation of Working Groups together with schools and residents of the surrounding neighborhoods.

A key innovation introduced by the NGO is the figure of the Community Teacher (Professor Comunitário). The Community Teacher plays the role of articulator in their territory with the task of developing initial diagnostics of the needs of the community and their resources, as well as identifying local leaders and creating networks. This innovation represents a new role within the education system that bridges formal schooling and community knowledge, facilitating the integration of territorial resources into educational processes.

The strong network developed by City-School-Apprentice constitutes one of its main innovations. These networks connect not only schools but also communities, researchers and local government, creating an ecosystem for educational innovation that operates across multiple levels. The organization's ability to articulate diverse actors around a shared educational vision demonstrates how social innovation in education requires sustained relationship-building and collaborative governance structures.

#### 5.3.2 Case 2: Campos Salles School

A relevant result of the different social innovations in education at Campos Salles School is the institutionalization of this innovation, which is visible in the strength of the relationship between Campos Salles School and the community of Heliópolis. This relationship has not only been strengthened through shared physical space but through the implementation of this school-community project. As stated in the school's Political Pedagogic Project of the own school:

“Today we are part of the Centre for Educational and Cultural Coexistence Heliópolis, a conquest of the organised community and the school.” (Political Pedagogic Project, 2014)

The key elements of innovation at Campos Salles School include the physical transformation of the school infrastructure into a community centre, the creation of the School Commission of Parents, Students and Teachers, and the development of specific assessment practices tailored to the needs of Heliópolis students. These innovations demonstrate how educational spaces can be reimagined as community assets and how pedagogical practices can be adapted to local contexts through participatory decision-making processes. The emphasis on building social capital within the community represents a fundamental innovation that connects educational improvement with broader community development goals.

#### 5.3.3 Case 3: INED (Institute of Educational Innovation)

This initiative has contributed significantly to diagnostic work and to developing experimental and pilot projects for teacher training in Chiapas. The key elements of innovation at INED include the use of participatory methods for diagnosis and decision-making processes, participatory approaches in training, and specific attention to the needs of indigenous teachers in rural schools in southern Mexico.

INED's evolution from a science dissemination centre to a teacher training institution represents an adaptive innovation responding to identified gaps in state provision. The organization's focus on the ethical; dignity, and emotional dimensions of teacher training, alongside fostering greater autonomy amongst teachers (Ulloa Reyes, 2022), represents an innovative approach that goes beyond traditional technical skills development. The institution's role as a reflexive and critical space, linking international and national institutions and resources, demonstrates how civil society organizations can serve as intermediaries that connect local educational needs with broader knowledge networks (Keck and Vázquez Sánchez, 2022). The case of INED demonstrates the importance of adapting to local needs and contexts in educational innovation, particularly in marginalized regions with indigenous populations. The evolution from science dissemination to teacher training illustrates how social innovations in education respond to identified gaps in state provision and how civil society organizations can play a crucial role in addressing educational inequalities in underserved communities. More evaluation and research is still needed to understand the full impact of this innovation and its current institutionalization process. However, this social innovation in education has represented an important step in recognizing the needs for providing more and better education not only to students but also to teachers, particularly in marginalized and indigenous communities where state support for professional development is limited.

### 5.4 Convergences and contextual differences across cases

Despite their distinct contexts, the three cases share several common features. In all cases, social innovation in education emerged from civil society initiatives responding to gaps in state provision, and all three rely on the active participation of local communities, civil society organizations, and the public sector. A further shared characteristic is the emphasis on collective and participatory approaches, whether through community-school integration in São Paulo or participatory teacher training in Chiapas.

However, the cases also reflect important contextual differences. City-School Apprentice (Case 1) operates in an urban city centre context, focusing on school-community integration through the use of public space, art, and culture to address social inequalities in a high-crime neighborhood. Campos Salles School (Case 2), situated in the urban periphery, centres its innovation on transforming the physical and pedagogical space of the school into a community asset, driven primarily by practitioners and community members without formal academic involvement. INED (Case 3), operating in a combination of urban-rural and predominantly indigenous context of Chiapas, focuses on teacher training and the inclusion of indigenous teachers and cultural traditions, addressing the specific challenges of marginalized rural and indigenous communities.

These differences reflect how social innovation in education is shaped by local socio-spatial contexts, available resources, and the specific educational inequalities each initiative seeks to address.

## 6 Discussion and limitations

### 6.1 Discussion

This research advances an understanding of social innovation in education by reflecting how innovation emerges, whose actors are involved, and in which stage. It specifically contributes to social innovation in education by showing how networks established by community-based organizations can evolve into long-term collaborative structures whilst maintaining their emphasis on community participation and co-creation. The variation between Brazilian and Mexican cases illustrates how contextual factors shape innovation forms and understandings, confirming that social innovation in education requires adaptation to specific historical, political, and cultural conditions rather than universal models.

Although the results of this research cannot be generalized, the findings contribute to a better understanding of the significant potential of social innovation in education to democratize educational systems by redistributing power and fostering greater autonomy among traditionally marginalized actors. The case studies show multiple mechanisms through which power relations are transformed: for example, the introduction of new actor roles such as the Community Teacher, the creation of participatory governance structures like the School Commission at Campos Salles School (Case 2), and the establishment of networks that connect communities directly with decision-making processes. These innovations challenge the hierarchical relationship between teachers and students, schools and communities, and central authorities and local actors that characterizes traditional educational systems (Torres, 2000). The emphasis on co-creation processes and participatory methods across all three cases reflects social change through innovations that alter not only educational practices but also the role of actors in the education system and in society (Maldonado-Mariscal, 2020). Although democratizing dynamics were not the original focus of this study, the findings suggest that, in the cases examined, community actors progressively moved from passive recipients to active participants in shaping educational practices. This shift resonates with Freire (1993 [1970]) conceptualization of educational processes that foster critical consciousness and social transformation, though such an interpretation should be understood as an analytical observation grounded in these specific contexts rather than a broader claim. Similarly, in all three cases, civil society organizations appeared to function as intermediaries connecting grassroots demands with formal institutions. This is consistent with what (Moulaert 2009) identifies as the potential of social innovation to address power imbalances, though the extent to which this translates into more just and democratic outcomes remains context-dependent and warrants further investigation. These findings invite dialogue with emerging literature on grassroots innovation in the Global South (Busacca and Coscarello, 2025; Maldonado-Mariscal, 2023; Maldonado-Villalpando et al., 2022). In particular, research from Mexico points to the emergence of institutionally embedded innovations oriented toward the territorial development of marginalized communities (Bucio-Mendoza and Solis-Navarrete, 2024), a dynamic that partly resonates with the patterns observed in the cases examined here, though the extent and conditions of such alignment require further comparative investigation.

### 6.2 Limitations

This study acknowledges some limitations. First, whilst primary fieldwork was conducted in 2013 and 2014, some intermediate developments may not have been fully captured, although the temporal distance allows for the observation of long-term impacts and sustainability. Second, the study presents a degree of asymmetry between the two national contexts: the focus group and the 2023 follow-up interviews were conducted exclusively in Brazil. However, both constituted supplementary data sources, secondary to the primary interview part. The comparative analysis between Brazil and Mexico rests on a set of semi-structured interviews conducted with parallel stakeholder categories in both countries. The additional Brazilian data served to contextualize and validate findings within that case, particularly given the researcher's greater familiarity with the Mexican context, and were not used to generate cross-case comparative claims. The asymmetry therefore reflects a difference in contextual depth rather than a structural imbalance in the comparative design. This is consistent with a most-different cases logic, in which Brazil and Mexico are treated as analytically distinct units selected for their divergence, with the aim of identifying innovation dynamics that persist across contrasting educational and institutional contexts. The focus on three case studies in specific regions, São Paulo in Brazil and Chiapas in Mexico, similarly limits generalisability, though this is an expected feature of qualitative comparative research, which prioritizes depth of understanding over breadth. Third, reliance on the INNOVEMOS platform for initial case identification may have excluded undocumented innovations. This limitation was partially addressed through exploratory fieldwork, unstructured interviews, and snowball sampling, which helped validate the representativeness of the selected cases and capture a broader network of actors. Despite these limitations, the study provides valuable empirical evidence of how social actors collaborate to construct and sustain social innovation in education in two distinct Latin American contexts.

## 7 Conclusions

This study addresses the following question: *How do social actors drive social innovation in education, and what role do networks, partnerships and community engagement play in these processes?*

The empirical evidence from three case studies in Brazil and Mexico reveals three main findings.

Firstly, developing strong networks and partnerships appears to be important for sustaining social innovation in education, as demonstrated by the cases examined. The City-School Apprentice (Case 1) illustrates this most clearly, having evolved from a local community organization in 1997 and having had a long-term impact on innovation in education. This trajectory suggests that, under favorable institutional conditions, grassroots initiatives may develop into larger collaborative structures while maintaining community participation. This is consistent with arguments about the role of civil society organizations as intermediaries between local educational needs and broader knowledge networks. However, the conditions enabling such scaling up remain highly context-dependent. It is worth noting, as a contextual observation rather than a causal claim, that at least two individuals interviewed as part of the City-School Apprentice have since become key actors in national initiatives such as (Escolas 2024a,b) and the Movement of Innovation in Education (Movimento de Inovação na Educação, 2022). This is not presented as evidence that the local initiative generated these programmes, nor as a basis for generalization. Rather, it serves as a small indicator that some of the actors embedded in locally rooted innovation practices in education have developed trajectories that connect them to broader national efforts oriented toward educational transformation and civil society engagement. This suggests, tentatively, that local and regional innovation ecosystems may, in certain conditions, function as spaces where actors develop the networks, knowledge, and legitimacy that later position them within wider coordinated strategies for educational improvement. Whether this pattern holds beyond this specific case remains an open question that warrants further investigation.

Second, community engagement emerges as a recurring feature across the three cases, though it takes different forms depending on the national and local context. Campos Salles School (Case 2) transformed its physical and pedagogical space into a community educational centre, whilst INED (Case 3) addressed the specific needs of indigenous teachers in marginalized contexts in Chiapas. In both cases, local communities were involved as active participants in shaping educational practice, which resonates with what (Schröder and Krüger 2019) describe as the capacity of social innovation to repair, modernize, or transform education systems, though the depth and sustainability of this participation varied considerably across contexts.

Third, understandings and conditions of innovation vary significantly by context. The Brazilian cases suggest the presence of denser institutional networks, with a wider range of stakeholders involved in educational policy development and greater continuity in programme implementation. In contrast, INED (Case 3) faced greater challenges due to limited resources and weaker institutional support, resulting in less continuity in network development. This contrast shows how local conditions, state support, and available resources shape both the form and sustainability of social innovation in education.

The main forms of innovation identified include organizational innovation through strengthening new learning processes within schools and NGOs, and social innovation in education through new school-community models (Case 2: Campos Salles School), new methods for empowerment and network development (Case 3: INED), and new partnerships amongst actors through participatory learning methods in the city and neighborhood (Case 1: City-Scholl Apprentice). Taken together, these findings suggest that, in the cases studied, social innovation in education emerged not as an isolated event but through iterative processes involving sustained relationship-building, negotiation between actors, and varying degrees of institutional support. Further research is needed to understand what role the state plays in fostering or hindering these processes, particularly in contexts where, as one Mexican interviewee noted, state recognition of innovation remains largely symbolic: “No, unfortunately, neither creativity nor innovation is stimulated. The prizes awarded by Institutions of Evaluation and Innovation are more of a symbolic nature” (State Representative and Researcher, Mexico).

The primary contribution of this research lies offering an empirically grounded account of social innovation in education in two Latin American contexts, contributing to a sociological understanding of new social practices in education, new forms of collaboration, the creation of new institutions, and the creation of alliances and networks of different actors who previously did not collaborate with each other (Maldonado-Mariscal and Schröder, 2023). The findings indicate that these processes operate primarily at the micro level, driven by the sustained involvement of educational and community actors, and that their form and reach are closely tied to the institutional and political conditions of each national context. This points to the need for further comparative research across a wider range of cases to assess the broader applicability of these patterns.

### 7.1 Outlook

The findings of this research point to several areas that warrant further investigation. The three cases examined suggest that civil society organizations could play a significant role in establishing new forms of collaboration between schools, communities, and other stakeholders. However, the conditions under which this occurs vary considerably across the Brazilian and Mexican contexts studied. The observed connections between these locally rooted practices and broader frameworks of grassroots innovation suggest the potential for a productive theoretical dialogue. However, further empirical work across a wider range of cases would be required before conclusions could be drawn about the significance of this link for educational and social transformation in Brazil and Mexico as a whole.

The trajectory of City-School Apprentice, which evolved from a local community organization into an involvement into national strategies raises questions about the conditions under which locally rooted initiatives develop connections to wider educational systems. This case suggests that scaling need not imply the loss of community participation, though it also illustrates that such trajectories require sustained institutional support, deliberate knowledge systematization, and the involvement of multiple stakeholders including universities, foundations, and, in some instances, government actors. Whether this trajectory is replicable in contexts with weaker institutional ecosystems, such as those observed in the Mexican case, remains an open and important question for future research.

Across the cases examined, the capacity of civil society organizations and educational institutions to build new partnerships and develop locally adapted practices appears closely tied to the availability of spaces for co-creation among teachers, community members, and institutional actors (Maldonado-Mariscal and Schröder, 2023). This is consistent with recent research suggesting that educational innovation tends to emerge through collaborative and institutional processes rather than as the product of top-down reform (Batista and Helal, 2023), though the extent and form of such collaboration differed notably between the Brazilian and Mexican cases in this study. These observations point to the value of further comparative research that examines how different national and institutional contexts shape the conditions under which such collaborative innovation can develop and be sustained, and what role, if any, state actors play in enabling or constraining these processes.

*Future research* should examine several important questions that emerge from, but extend beyond, the scope of this study. First, how can networks and partnerships developed within locally rooted educational initiatives be sustained over time, particularly where state support is limited? Second, what institutional arrangements may facilitate scaling of local innovations whilst preserving their community-centred character? Third, how can universities better support locally rooted educational initiatives through action-research partnerships that generate knowledge with and for communities? (Maldonado-Mariscal and Kriegel, 2026). Comparative research across a wider range of Latin American contexts would strengthen understanding of how political, economic, and cultural conditions shape social innovations in education. The two countries examined in this study offer contrasting institutional landscapes, yet both are marked by significant inequalities in access to educational resources and persistent tensions between centralized policy and local community needs. Research extending to other national contexts characterized by similar structural conditions could help clarify which patterns observed here are context-specific and which may recur more broadly (Busacca and Coscarello, 2025). The case of INED in Chiapas also points to the relevance of autonomous and indigenous educational traditions as distinct sites of innovation. Research in this area, including work on Zapatista education, suggests that some locally rooted initiatives generate not only new pedagogical practices but also alternative frameworks for understanding the relationship between education and community development (Maldonado-Villalpando et al., 2022). This dimension remains underexplored in comparative educational research and warrants further attention. The role of the state in fostering or hindering innovation requires deeper examination. Whilst this study suggests that social innovations can emerge with limited state support, long-term sustainability may be shaped by enabling policy environments that recognize the agency of teachers, communities, and civil society organizations. Future research should explore how public policies can support social innovation without imposing standardized models that undermine contextual adaptation and community participation. Further research should also examine the relationship between social movements and social innovation in education. Across the cases studied, there were indications that social innovations emerged through iterative processes involving collective action and, in some instances, social contestation, a pattern consistent with existing theoretical work in this area (Maldonado-Mariscal, 2020; da Gohn and Maldonado-Mariscal, 2023). In at least some of the cases examined, actor trajectories appeared to shift over time, moving from locally embedded mobilization toward more formalized roles connecting schools, communities, and institutional partners. This evolution represents the maturing and professionalization of knowledge within social innovation (da Gohn and Maldonado-Mariscal, 2023). The changing role of universities warrants investigation, particularly as higher education institutions face demands for community engagement (Cobo-Gómez, 2024) whilst navigating internal resistance to forms of engagement (Clara Zafra et al., 2025) that challenge established institutional logics (Benneworth and Cunha, 2015; Loorbach and Wittmayer, 2024). Whilst this study did not focus directly on the role of universities, the cases suggest that higher education actors were present, to varying degrees, in the networks surrounding the initiatives examined. Further research could usefully explore how universities position themselves in relation to locally rooted educational innovation, and under what conditions they function as genuine partners in knowledge co-creation rather than as external evaluators or knowledge extractors.

## Data Availability

The datasets presented in this article are not readily available because the raw data is stored locally and can be provided as exemplary cases upon request. However, all data must be anonymised. Requests to access the datasets should be directed to karina.maldonado@tu-dortmund.de.
